# Comparison of sCIM and Other Phenotypic Detection Methods for Carbapenemase-Producing *Enterobacterales*

**DOI:** 10.1128/Spectrum.01608-21

**Published:** 2021-11-17

**Authors:** Takuya Hosoda, Yohei Doi, Masahiro Suzuki

**Affiliations:** a Department of Clinical Laboratory, Fujita Health University Okazaki Medical Center, Okazaki, Japan; b Department of Microbiology, Fujita Health University School of Medicine, Toyoake, Japan; c Department of Infectious Diseases, Fujita Health University School of Medicine, Toyoake, Japan; d Division of Infectious Diseases, University of Pittsburgh School of Medicine, Pittsburgh, Pennsylvania, USA; Keck School of Medicine of the University of Southern California

**Keywords:** carbapenemase-producing *Enterobacterales*, sCIM, mCIM

## Abstract

Rapid detection and reporting of carbapenemase-producing *Enterobacterales* (CPE) is one of the top priorities of clinical microbiology laboratories. The Clinical and Laboratory Standards Institute recommends the modified carbapenem inactivation method (mCIM) as the preferred method for this purpose, but it requires a broth incubation process which can be cumbersome. Here, we compared the performance of mCIM with three alternative rapid CPE detection methods against a collection of genetically defined CPE, with most carrying *bla*_IMP_, and non-CPE clinical isolates. The sensitivities of mCIM, simplified carbapenem inactivation method (sCIM), Rapidec Carba NP, and NG-Test Carba 5 were 98.0%, 54.9%, 90.2%, and 72.5%, whereas the specificities were 89.5%, 84.2%, 89.5%, and 100%, respectively. Modification of the interpretive criteria of sCIM increased its sensitivity to 88.2% and specificity to 89.5%. The results suggest that mCIM is currently the optimal method for CPE detection in an epidemiological setting where CPE-producing IMP group carbapenemase is predominant. While sCIM is easier to perform, it requires further validation before it can be widely adopted as an alternative to mCIM in the clinical laboratory.

**IMPORTANCE** Simple identification methods for carbapenemase-producing *Enterobacterales* are required for the clinical laboratory. The simplified carbapenem inactivation method (sCIM) is a carbapenemase detection method that can be performed with less hands-on time than mCIM, but its sensitivity and specificity were suboptimal compared with other phenotypic detection methods when tested against a collection of IMP-producing CPE. Insufficient inactivation of imipenem from inadequate inoculation was suspected as the cause. While sCIM is easier to perform, it requires optimization before it can be widely adopted as an alternative to mCIM in the clinical laboratory.

## INTRODUCTION

Carbapenem-resistant *Enterobacterales* (CRE) are some of the top priority antimicrobial-resistant pathogens which are associated with few treatment options and suboptimal clinical outcomes for infected patients (1). CRE isolates that produce carbapenemase (carbapenemase-producing *Enterobacterales* [CPE]) are particularly problematic as they can spread easily among patients and the carbapenemase genes carried on plasmids may be transmitted among bacteria and cause multispecies outbreaks (2). Rapid and accurate detection of CPE in the clinical laboratory is therefore of paramount importance.

While PCR is the gold standard for the detection of carbapenemase genes, it requires dedicated equipment and reagents as well as training of the laboratory personnel. Additionally, the significant hands-on time and challenges with quality control make it difficult to implement it in a standard clinical laboratory. The Clinical and Laboratory Standards Institute (CLSI) recommends the modified carbapenem inactivation method (mCIM) as the preferred detection method for CPE (3). mCIM has been adopted broadly by clinical laboratories as it detects isolates with modest carbapenemase activity and can be performed with supplies that are routinely available in clinical laboratories. However, the need to incubate the isolates with a meropenem disk in broth for several hours can make it difficult to complete the process within regular work hours. The simplified carbapenem inactivation method (sCIM), which eliminates the broth incubation process, has been proposed as an alternative, nonproprietary CPE detection method (4). Reports on sCIM remain scarce, however, and its performance has not been validated thoroughly.

Additionally, several phenotypic detection methods of CPE are commercially available for use in the clinical laboratory. One such method is Rapidec Carba NP (bioMérieux) which enables the detection of carbapenemase activity from an isolated colony within 30 minutes by leveraging pH changes (5). Another method with high sensitivity and specificity is NG-Test Carba 5 (NG Biotech), which is an immunochromatographic assay that detects specific carbapenemase groups in 15 minutes (6).

The aim of this study was to compare the performance of sCIM and mCIM as well as two rapid CPE detection methods (Rapidec Carba NP, and NG-Test Carba 5) against well-defined CPE and non-CPE clinical isolates in terms of sensitivity, specificity, ease of process and interpretation, and feasibility in the clinical laboratory.

## RESULTS

The results are summarized in Table 1.

**TABLE 1:** Sensitivity and specificity of the CPE detection methods

Organism	Values for^*a*^:				
	mCIM	sCIM	sCIM (alternative criteria)	CARBA NP	CARBA 5
Carbapenemase-producing *Enterobacterales* by enzyme (*n*)					
IMP-1 (48)					
Enterobacter cloacae complex	20/20	13/20	19/20	18/20	15/20
Escherichia coli	1/1	1/1	1/1	1/1	1/1
Klebsiella michiganensis	16/16	7/16	15/16	15/16	13/16
Klebsiella pneumoniae	6/7	2/7	3/7	6/7	2/7
Providencia rettgeri	1/1	0/1	1/1	1/1	1/1
Serratia marcescens	3/3	2/3	3/3	3/3	3/3
NDM-5 (1)					
Escherichia coli	1/1	1/1	1/1	1/1	1/1
OXA-48 (1)					
Escherichia coli	1/1	1/1	1/1	0/1	1/1
IMI-2 (1)					
Enterobacter bugandensis	1/1	1/1	1/1	1/1	0/1
Total	50/51	28/51	45/51	46/51	37/51
Sensitivity (%)	98.0	54.9	88.2	90.2	72.5
Non-carbapenemase-producing *Enterobacterales*					
Citrobacter freundii	1/1	1/1	1/1	1/1	1/1
Klebsiella aerogenes	4/4	4/4	3/4	4/4	4/4
Enterobacter cloacae complex	5/5	4/5	4/5	5/5	5/5
Escherichia coli	2/3	2/3	3/3	2/3	3/3
Klebsiella pneumoniae	2/2	2/2	2/2	2/2	2/2
Serratia liquefaciens	1/1	1/1	1/1	1/1	1/1
Serratia marcescens	1/2	1/2	2/2	1/2	2/2
Providencia rettgeri	1/1	1/1	1/1	1/1	1/1
Total	17/19	16/19	17/19	17/19	19/19
Specificity (%)	89.5	84.2	89.5	89.5	100

a All values are n/total except where indicated.

### mCIM and sCIM.

The sensitivity and specificity of mCIM were 98.0% (50/51) and 89.5% (17/19), respectively. There were 3 isolates for which the results were indeterminate. On the other hand, the sensitivity and specificity of sCIM were much lower at 54.9% (28/51) and 84.2% (16/19) with 10 indeterminate isolates. All 20 isolates for which sCIM gave false-negative results were IMP-1-producing isolates. When we modified the interpretive criteria to a zone of inhibition of ≤24 mm and ≥25 mm as positive and negative, respectively, the sensitivity increased to 88.2% (45/51) and the specificity also improved to 89.5% (17/19).

### Rapidec Carba NP.

The sensitivity and specificity of Rapidec Carba NP were 90.2% (46/51) and 89.4% (17/19), respectively, whereas false-positive and false-negative results accounted for 10.5% (2/19) and 9.8% (5/51), respectively. The false-negative results occurred in 4 IMP-1- and 1 OXA-48-producing isolates.

### NG-Test Carba 5.

The sensitivity and specificity of NG-Test Carba 5 were 72.5% (37/51) and 100% (19/19), respectively. The carbapenemase groups predicted by the assay were fully congruent with the carbapenemase genes identified by whole-genome sequencing. The false-negative results consisted of 13 IMP-1- and 1 IMI-2-producing isolates. IMI group carbapenemases are not detected by this assay.

## DISCUSSION

CPE are not only resistant to carbapenems but also typically resistant to other classes of agents, and thus, infections caused by these isolates are difficult to treat (1). Furthermore, the carbapenemase genes are located mostly on plasmids, enabling them to move across strains and species (2). Given their clinical and epidemiological importance, infections caused by CRE, including CPE, became reportable in Japan in 2014. Rapid and accurate detection of CPE is critical for informing appropriate therapy and also preventing its spread in the hospital.

We conducted this study to determine the performance of various phenotypic CPE detection methods that are feasible in clinical laboratories, including the recently reported sCIM. We found that mCIM, the most widely implemented CPE detection method at this time, provided excellent sensitivity and specificity. Importantly, no CPE isolate was erroneously categorized as non-CPE. In addition, all 50 positive isolates gave a zone diameter of 6 mm, i.e., no zone of inhibition, making it easy for the technologists to interpret the results. mCIM has its own shortcomings, such as the need for a broth incubation process followed by overnight incubation, but the procedure and interpretation are generally straightforward, thus reducing the risk of intertechnologist variability.

**FIG 1:**
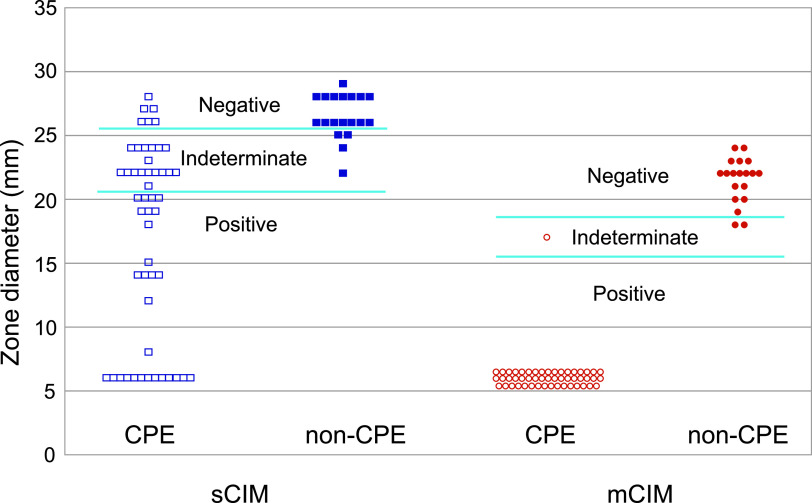
Zone diameter of CPE and non-CPE isolates with sCIM and mCIM assays. The square and round points correspond to sCIM and mCIM, respectively. The white and filled points correspond to CPE and non-CPE, respectively.

sCIM was proposed by Jing and colleagues with the goal of simplifying mCIM. sCIM bypasses the 4-hour broth incubation process which makes it easier to incorporate the procedure in the routine workflow of the laboratory (4). Also, the better performance of sCIM in detecting metallo-beta-lactamases and a shorter time to result compared with mCIM has been demonstrated by Baeza et al. (7). In our hands, sCIM could consistently be set up within the typical 8-hour work day. However, we also observed several shortcomings. First, the inoculum is difficult to standardize since the morphology and size of the originating colonies are highly variable. In fact, the sensitivity of sCIM has been reported to depend significantly on the inoculum used (8). On a related note, we found that manual application of bacteria onto the imipenem disk could be technically challenging and carried the risk of contaminating the laboratory environment. Third, unlike mCIM where CPE isolates produced little or no zone of inhibition, sCIM tended to give larger zones of inhibition even for CPE isolates, often making it difficult to distinguish between CPE and non-CPE isolates, as shown in Fig. 1. Fourth and foremost, the sensitivity and specificity were both low at 54.9% and 84.2%, respectively. These rates were much lower than those reported in the original description of sCIM. While the reasons for this discrepancy are unclear, one possibility is that imipenem in the disks was not sufficiently hydrolyzed in our setting. In fact, when we modified the cutoff values to accommodate CPE isolates with larger zone sizes by eliminating the indeterminate category, both the sensitivity and specificity improved. We conclude that sCIM requires further validation and improvement before it can be recommended as a preferred alternative to mCIM in the clinical laboratory.

Rapidec Carba NP uses a color indicator to detect pH change due to hydrolysis of imipenem and has the advantage of providing results within 2 hours total without the need for overnight incubation (5). However, the procedure is more complicated than mCIM or sCIM, and the interpretation is qualitative based on visual inspection of the color of the buffer. This characteristic could make using Rapidec Carba NP problematic in regions, including Japan, where IMP group enzymes with relatively low carbapenemase activity predominate (9). The sensitivity of this method for isolates producing another carbapenemase with weak activity, namely, OXA-48, is also reported to be limited (10). The only OXA-48 producer in our study gave a false-negative result, supporting this concern.

Enzyme-specific immunoassays have the advantage of distinguishing the carbapenemase groups, thus potentially informing hospital epidemiology and treatment choices. NG-Test Carba 5 is one such immunochromatographic assay that detects the production of KPC, OXA-48, VIM, IMP, and NDM group carbapenemases (6). The gold standard assay to distinguish carbapenemase groups is PCR, but NG-Test Carba 5 has the advantage of not requiring any equipment as would be for PCR and thus provides a solid alternative to PCR when the information on specific carbapenemase can be useful. The assay was also easy to perform and provided results in 20 minutes total. One major caveat of NG-Test Carba 5 is its variable sensitivity for IMP-producing CPE isolates, which was 72.5% in our study and ranges between 55.6% and 100% in the literature (7, 9). Since we used strains detected in a single hospital, it is possible that many of the strains happened to be more likely to give a false-negative result by Carba 5. In regions where IMP predominates among CPEs, additional testing with another method like mCIM may be considered when suspicion for CPE is high but the result of NG-Test Carba 5 is negative, at least until a revised assay with improved detection of this carbapenemase group becomes available (11). From the local perspective, the high cost of NG-Test Carba 5 may also limit uptake by clinical laboratories, as it is not approved for reimbursement in Japan at the time of this writing.

Our study is limited by a relatively small number of CPE isolates tested, of which most were IMP-1-producing isolates collected at a single hospital. In addition, each test was run once in the clinical laboratory, and thus, the reproducibility of each method could not be confirmed. However, it is reflective of the current molecular epidemiology of CPE in Japan, and the performance of the assays tested here are likely to be applicable locally.

In conclusion, we compared the performance, ease of procedure, and interpretation and turnaround time of four CPE detection methods that can be implemented in clinical microbiology laboratories using a panel of CPE clinical isolates at a hospital in Japan. We suggest that sCIM requires further improvement before application in the clinical laboratory, and that mCIM remains the optimal method overall especially where IMP producers are common among CPE.

## MATERIALS AND METHODS

### Bacterial isolates.

A total of 70 unique CRE clinical isolates archived at the clinical microbiology laboratory of Fujita Health University Hospital were used (Table 2). Each isolate was collected from a different patient except for two non-CPE, CRE isolates (Escherichia coli and Serratia marcescens), which were collected from the same patient. The whole genome of all isolates, except for one Klebsiella pneumoniae CPE isolate, were sequenced by MiSeq or NextSeq 2000 instruments using the NexteraXT DNA library preparation kit or QIAseq FX DNA library kit (Qiagen, Hulsterweg, Netherlands) and MiSeq reagent kit v3 600 cycles or NextSeq 1000/2000 P2 reagents 200 cycles (Illumina, San Diego, CA). Sequence reads were assembled using SPAdes v3.13.1. The remaining isolate was sequenced by a MinION device using the ligation sequencing kit SQK-LSK109 (Oxford Nanopore Technologies, Oxford, UK). Sequence reads were assembled using Flye v2.7. Carbapenemase genes were detected in 51 of the 70 isolates and included *bla*_IMP-1_ (*n* = 48), *bla*_OXA-48_ (1), *bla*_NDM-5_ (1), and *bla*_IMI-2_ (1). The remaining 19 isolates were non-CPE, CRE isolates. Antimicrobial susceptibility testing was conducted on a Vitek 2 instrument using the AST-N229 card (bioMérieux).

**TABLE 2:** CRE clinical isolates used in the study

Organism	MIC range (mg/liter) of:						
	*n*	MEM	IPM	CAZ	ATM	TZP	CMZ
Carbapenemase-producing *Enterobacterales* by enzyme (*n*)							
IMP-1 (48)							
Enterobacter cloacae complex	20	4 to >8	1 to >8	>32	≤1 to >32	≤4 to >64	>32
Escherichia coli	1	>8	>8	>32	>32	>64	>32
Klebsiella michiganensis	16	≤0.25 to >8	1 to >8	16 to >32	≤1 to >32	8 to >64	32 to >32
Klebsiella pneumoniae	7	≤0.25 to >8	≤0.25 to >8	16 to >32	≤1 to 16	8 to >64	32 to >32
Providencia rettgeri	1	8	4	>32	≤1	≤4	>32
Serratia marcescens	3	4 to >8	4 to >8	>32	≤1	≤4	>32
NDM-5 (1)							
Escherichia coli	1	8	>8	>32	≤2	>64	>32
OXA-48 (1)							
Escherichia coli	1	≤0.25	≤0.25	>32	16	>64	8
IMI-2 (1)							
Enterobacter bugandensis	1	>8	>8	≤1	≤1	≤4	>32
Non-carbapenemase-producing *Enterobacterales*							
Citrobacter freundii	1	0.25	4	≤1	≤1	≤4	>32
Klebsiella aerogenes	4	0.25 to >8	2 to >8	≤1 to >32	≤1 to >32	≤4 to >64	>32
Enterobacter cloacae complex	5	0.25 to 4	1 to 4	≤1 to >32	≤1 to >32	≤4 to >64	>32
Escherichia coli	3	0.25 to 8	2 to 8	4 to >32	16 to >32	>64	>32
Klebsiella pneumoniae	2	4 to 8	1 to 2	16 to >32	>32	>64	32 to >32
Serratia liquefaciens	1	4	4	4	4	>64	>32
Serratia marcescens	2	≤0.25 to 4	2 to 8	≤1 to >32	≤1 to >32	64 to >64	>32
Providencia rettgeri	1	4	1	≤1	≤1	≤4	>32

### mCIM.

mCIM was performed according to the document M100-S27 by the CLSI (3). A zone of inhibition of ≤15 mm, 16 to 18 mm, and ≥19 mm around the meropenem disk was interpreted as positive, indeterminate, and negative, respectively.

### sCIM.

For sCIM, one to three colonies that grew on a tryptic soy blood agar plate were applied on an imipenem disk (BD). The disk was then placed on a Mueller-Hinton agar plate inoculated with Escherichia coli ATCC 25922 and incubated at 35°C aerobically overnight, and the zone of inhibition around the imipenem disk was measured. A zone of inhibition of ≤20 mm, 21 to 25 mm, and ≥26 mm was interpreted as positive, indeterminate, and negative, respectively (4). When colonies were observed within the zone of inhibition inside the 20-mm cutoff, the test was interpreted as positive.

### Rapidec Carba NP.

Carba NP was performed according to the manufacturer’s instructions. The results were visually interpreted 30 minutes after the reaction, where yellow, yellow-orange, and orange were interpreted as positive and red-orange and red as negative. For indeterminate results, incubation was extended by an hour at 35°C and interpreted again as per the manufacturer’s recommendation.

### NG-Test Carba 5.

NG-Test Carba 5 was conducted according to the manufacturer’s instructions. Briefly, approximately three colonies on a blood agar plate were suspended in 150 μL of the designated extraction buffer, and 100 μL of the mixture was applied to the sample well. The results were read after 15 minutes.

All tests were performed once.

### Data availability.

Sequence reads of the strains are available under the BioSample accession numbers SAMN21893121 to SAMN21893190.
